# Familial Occurrence of Systemic Mast Cell Activation Disease

**DOI:** 10.1371/journal.pone.0076241

**Published:** 2013-09-30

**Authors:** Gerhard J. Molderings, Britta Haenisch, Manuela Bogdanow, Rolf Fimmers, Markus M. Nöthen

**Affiliations:** 1 Institute of Human Genetics, University Hospital of Bonn, Bonn, Germany; 2 German Center for Neurodegenerative Diseases (DZNE), Bonn, Germany; 3 Federal Institute for Drugs and Medical Devices (BfArM), Bonn, Germany; 4 Department of Psychiatry, University of Bonn, Bonn, Germany; 5 Institute for Medical Biometry, Informatics and Epidemiology, University Hospital of Bonn, Bonn, Germany; University of Birmingham, United Kingdom

## Abstract

Systemic *mast cell activation disease* (MCAD) comprises disorders characterized by an enhanced release of mast cell mediators accompanied by accumulation of dysfunctional mast cells. Demonstration of familial clustering would be an important step towards defining the genetic contribution to the risk of systemic MCAD. The present study aimed to quantify familial aggregation for MCAD and to investigate the variability of clinical and molecular findings (e.g. somatic mutations in KIT) among affected family members in three selected pedigrees. Our data suggest that systemic MCAD pedigrees include more systemic MCAD cases than would be expected by chance, i.e., compared with the prevalence of MCAD in the general population. The prevalence of MCAD suspected by symptom self-report in first-degree relatives of patients with MCAD amounted to approximately 46%, compared to prevalence in the general German population of about 17% (p<0.0001). In three families with a high familial loading of MCAD, the subtype of MCAD and the severity of mediator-related symptoms varied between family members. In addition, genetic alterations detected in *KIT* were variable, and included mutations at position 816 of the amino acid sequence. In conclusion, our data provide evidence for common familial occurrence of MCAD. Our findings observed in the three pedigrees together with recent reports in the literature suggest that, in familial cases (i.e., in the majority of MCAD), mutated disease-related operator and/or regulator genes could be responsible for the development of somatic mutations in KIT and other proteins important for the regulation of mast cell activity. Accordingly, the immunohistochemically different subtypes of MCAD (i.e. mast cell activation syndrome and systemic mastocytosis) should be more accurately regarded as varying presentations of a common generic root process of mast cell dysfunction, than as distinct diseases.

## Introduction

Systemic *mast cell activation disease* (MCAD) comprises disorders characterized by an enhanced release of mast cell mediators accompanied by accumulation of dysfunctional mast cells ([1] and references therein). According to current classification [1], [2], three subtypes of systemic MCAD are distinguished: *systemic mastocytosis* (SM); *mast cell activation syndrome* (MCAS); and *mast cell leukemia* (MCL). SM is characterized by specific pathological somatic mutations in exon 17 of the tyrosine kinase KIT (for which *KIT* ^D816V^ accounts for the great majority; reviewed in [3]) and immunohistochemical findings (known as the *World Health Organization (WHO) criteria;* [4]) caused by these mutations. A diagnosis of MCAS [1], [2], [5]–[7] is assigned to patients who present with multiple mast cell mediator-induced symptoms, but who do not fulfill the requirements for a diagnosis of SM, and in whom relevant differential diagnoses have been excluded. MCL is an aggressive mast cell neoplasm, defined by increased numbers of mast cells in bone marrow smears (≥ 20%) and the presence of circulating mast cells (reviewed in [4]). Recently, a prevalence of 1 in 364,000 for SM in Europeans has been calculated [3]. Data from a clinical population suggest that the prevalence of MCL is two orders of magnitude lower than that of SM [8]. In contrast to these rare forms of systemic MCAD, preliminary evidence suggests that MCAS is much more common in the population and may be an underlying cause of various clinical presentations (e.g., in subsets of patients with fibromyalgia and irritable bowel syndrome) and prevalence is hence likely to lie at least within the single-digit percentage range ([3]; and references therein).

Very little is known about the familiality of systemic MCAD. To date, only five familial cases (three SM and two MCAS) have been reported [9]–[13]. A systematic investigation of familial clustering would be an important step towards defining the contribution of inherited genetic factors to the risk of systemic MCAD. In a preliminary analysis of family data [3], solely based on information provided by index patients, we obtained first evidence that familial occurrence of MCAD might be a frequent phenomenon. In the present systematic family study we obtained direct information from first-degree relatives of MCAD patients and compared this to data obtained from the general population with the aim of quantifying the familial aggregation of MCAD. In addition, three multiply affected families were investigated in detail to determine the spectrum of clinical affections among family members as well as the variation in *KIT* muations and KIT isoforms.

## Methods

### Subjects

One-hundred-and-twenty-four patients ( =  index patients), who presented to our *Bonn Interdisciplinary Research Group for Systemic Mast Cell Diseases* between May, 2005, and November, 2011, for diagnostic reasons, were asked to participate in this study and 84 index patients with MCAD (MCAS n = 69, SM n = 15) agreed (Table 1, Table S1). In the index patients SM was diagnosed according to the World Health Organization criteria for definition of SM [4]. Diagnosis of MCAS was based on the proposed criteria to define MCAS [1], i.e. the typical clinical symptoms recorded in a standardized manner by our validated questionnaire [14], [15], exclusion of relevant differential diagnoses of MCAD which may present with organ- or tissue-related mast cell mediator-induced symptoms, determination of pathologically increased release of specific mast cell mediators, i.e. tryptase, heparin and histamin in blood and N-methylhistamine in urine, and in some cases detection of functionally activating genetic changes in mast cells. All patients were asked to obtain consent from first-degree relatives to allow us to contact them. After consent was obtained, first-degree relatives were contacted and asked to complete a questionnaire. Two-hundred-and-fifty-five first-degree relatives were enrolled (Table 1, Table S1). There were no monozygotic twins among the sample of index patients and first-degree relatives. A sample of 266 subjects (those who responded from a total of 1,000 persons contacted; Table S1), randomly recruited from the German inhabi­tants of the city of Bonn through addresses provided by the city’s registration office, served as a control group representative of the German population. Information on the ethnicity of all participants was obtained through documenting the country/town of origin of the grandparents of participants. The characteristics of the study population are listed in Table 1.

**Table 1:** Characteristics of index patients, their first-degree relatives, and healthy controls.

	Index patients	First-degree relatives	Healthy controls
	(n = 84)	(n = 255)	(n = 266)
**Variables**	**n (%)**	**n (%)**	**n (%)**
**Females**	62 (74%)	142 (56%)	143 (54%)
**Age (mean ± SD,**	48.3±13.8	52.1±20.6	52.7±18.3
**range)**	21–84	8–98	20–92
**Age groups (years)**			
**< 21**	0 (0%)	19 (8%)	1 (0.4%)
**21–39**	23 (27%)	51 (20%)	71 (27%)
**40–49**	23 (27%)	35 (14%)	48 (18%)
**50–59**	21 (25%)	38 (15%)	48 (18%)
**60–69**	10 (12%)	52 (20%)	46 (17%)
**70–79**	5 (6%)	36 (14%)	29 (11%)
**> 80**	2 (2%)	24 (9%)	23 (9%)
		Parents: 98	
		mother 60 (61%)	
		father 38 (39%)	
		Siblings: 94	
		sister 52 (55%)	
		brother 42 (45%)	
		Children: 63	
		daughter 30 (48%)	
		son 33 (52%)	

### Diagnostic procedure

In all relatives and control participants, a diagnosis of MCAD was judged likely, for purposes of our analyses, based on information documented in a self-report questionnaire (Table S2). This questionnaire was compiled on the basis of our previously published validated questionnaire [14], [15]. After exclusion of relevant differential diagnoses MCAD was diagnosed, if (1) 11 or more items of the questionnaire were applicable or (2) if potentially mast cell mediator-related symptoms occurred in five or more different organs and/or tissues. The information documented in the self-report questionnaire did not allow assignment of MCAD diagnosis to the subtypes SM and MCAS, because no data on mediator levels in blood or urine, or immunohistological or molecular findings from biopsies were recorded, and these are required for MCAD subclassification. In the three multiply affected pedigrees in which we characterized somatic *KIT* mutations and expression of KIT isoforms, severity of the MCAD was defined as recently proposed (mild: no drug therapy required; moderate: drug therapy required, but no need for hospitalization; severe: drug therapy and recurrent hospitalization required; [16]).

### Ethics statement

The study was approved by the Ethics Committee of the medical faculty of the Rheinische Friedrich-Wilhelms-University of Bonn (no. 260/07). All participants were informed in detail about the purpose of the study. All index patients and their relatives gave written informed consent prior to the investigation, according to the guidelines of the Ethics Committee. Due to the anonymous nature of the survey of the control population, the Ethics Committee waived the need for written informed consent from the control participants.

### Statistical analyses

All data were statistically analyzed using SAS version 9.2. Logistic regression was used to calculate odds ratios and 95% confidence intervals for the association of MCAD with mast cell-related symptoms and findings. Chi-square analysis was used to compare categorical data to obtain estimates of possible association. A P value <0.05 was considered to be statistically significant.

### Analysis of somatic KIT mutations and KIT isoforms in three selected families

Peripheral blood obtained by venipuncture was drawn into syringes containing EDTA as an anti-coagulant. Hematopoietic mast cell-committed progenitors were isolated as described previously [17]. Briefly, mast cell committed-progenitors were separated by density gradient centrifugation using FicollPaque Plus (GE Healthcare Bio-Sciences, Uppsala, Sweden) followed by immunomagnetic positive selection with mAb against human CD117 (the tyrosine kinase, KIT, encoded by the gene *KIT*) according to the manufactureŕs instructions (Magnetic cell sorting, MACS; Miltenyi Biotech, Bergisch Gladbach, Germany). RNA was extracted and reverse-transcribed by standard techniques. There was no contamination of genomic DNA in the RNA preparation as proved by control PCRs using primers enclosing intron-exon-boundaries. Specific PCR primers were designed to amplify overlapping fragments of corresponding *KIT* cDNAs. The primer sequences (Table S3) were adapted from the human *KIT* reference sequence (accession number NM_000222). PCR was carried out using cDNA, Failsafe PCR buffer G (Epicentre, Madison, WI, USA) and 1 U of Taq DNA Polymerase (Invitrogen, Karlsruhe, Germany), in a final volume of 50 µl. The PCR conditions were 40 cycles of 1 min denaturation at 94°C, 1 min at the appropriate annealing temperature for the primers (Table S3), and 1 min extension at 72°C, followed by a final 5 min extension period at 72°C. PCR products were separated by gel electrophoresis on a 1.8% agarose gel. The bands of interest were cut out of the gel, and the DNA was extracted using the MinElute Gel Extraction Kit (Qiagen, Hilden, Germany). PCR products were directly sequenced at Qiagen (Hilden, Germany).

The expression intensities of two splicing isoforms of transcripts of *KIT*, which are characterized by the presence or absence of the tetra­peptide sequence glycine-asparagine-asparagine-lysine (GNNK, amino acids 510–513) in the extracellular part of the juxtamembrane region were determined by agarose gel scanning densitometry. The ratios of the expression intensity of the splicing isoforms with the deletion of the amino acids 510–513 (GNNK(–)) over that without this deletion (GNNK(–)/⊕) were calculated.

## Results

A total of 84 families were studied. Of the index patients, 62 were female (74%) and 22 were male (26%), with a female to male ratio of 2.8:1 (Table 1). The age of the patients ranged from 21 to 84 years, with a mean age of 48.3 years (Table 1). The 84 index patients had a total of 372 first-degree relatives, of which 255 completed the questionnaire (Table S1). A total of 117 relatives could not be questioned for various reasons, including that they were no longer alive, or had no known contact details (Table S1). The groups of index patients, first-degree relatives, and healthy controls did not differ significantly in age distribution (Table 1).

### Familial occurrence of MCAD

Approximately 74% of our index patients had at least one first-degree relative with systemic MCAD, regardless of systemic MCAD subtype and gender (not shown). The prevalence of systemic MCAD among first-degree relatives in our sample was 46% (Table 2A), which differed significantly (p<0.0001) from the prevalence in the control group (approximately 17%; Table 2A). There was no significant difference in prevalence of MCAD among first-degree relatives of patients with the MCAD subtypes, MCAS and SM, at 60% and 44%, respectively (p = 0.1048). A female prepronderance was observed in affected relatives and controls (67%; 64%) which was slightly less pronounced than in the index patients (74%). The prevalence of MCAD was similar among the different groups of first-degree relatives (parents, siblings and children) (Table 2B).

**Table 2:** Prevalence of MCAD in first-degree relatives and healthy subjects (A) and within the first-degree relatives group (B).

	Diagnosis		
**(A)**	no MCAD	MCAD	Sum
Relatives	n = 13854.12%	n = 11745.88%	n = 255
Controls	n = 22183.08%	n = 4516.92%	n = 266
Sum	n = 359	n = 162	n = 521
Chi^2^-Test	p<0.0001		
**(B)**			
Parents	n = 4950.00%	n = 4950.00%	n = 98
Siblings	n = 5356.38%	n = 4143.62%	n = 94
Children	n = 3657.14%	n = 2742.86%	n = 63
Sum	n = 138	n = 117	n = 255
Chi^2^-Test	p = 0.5782		

The data in Tables 3 and 4 demonstrate that the prevalence of almost all symptoms and findings investigated by the questionnaire was significantly higher in first-degree relatives (Table 4) compared to controls with the prevalences lying between patients (Table 3) and controls (Tables 3 and 4).

**Table 3:** Odds ratios for the symptoms and findings collected in the questionnaire (independent variables) occurring in patients, compared with the healthy control group.

	Patients vs. controls			
	p-value	Odds ratio	95%-confidence interval	
Female gender	0.0011	2.424	1.4	4.2
Smoker	-			
Abdominal pain	<.0001	167.875	57.1	493.4
Diarrhea	<.0001	625.000	82.0	5000.0
Gastritis	<.0001	768.923*		
Meteorisms	<.0001	258.462*		
Nausea	<.0001	86.333	38.9	191.5
Non-cardiac chest pain	<.0001	85.483	37.7	193.7
Hypercholesterinemia	<.0001	4.310	2.5	7.3
Paresthesia/pain	<.0001	93.646	38.8	225.8
Tachycardia	<.0001	48.438	21.0	111.7
Flush	<.0001	152.269	61.5	376.9
Hot flash	-			
Blood pressure changes (acute)	<.0001	44.540	21.3	93.1
Thyroid dysfunction	<.0001	11.236	2.9	43.9
Diabetes mellitus	0.1386	4.882	0.5	49.5
Asthenia	<.0001	1583.385	204.0	12288.3
Fatigue	<.0001	79.757	32.4	196.2
Weight loss	<.0001	78.891	31.2	199.4
Angioedema	<.0001	288.000	32.5	2554.2
Conjunctivitis	<.0001	16.428	8.5	31.6
Tinnitus	<.0001	7.724	4.3	13.8
Nasal congestion/rhinorrhea	<.0001	11.455	6.2	21.3
Pulmonary symptoms	<.0001	52.464	22.4	122.6
Headache	<.0001	26.924	13.5	53.9
Urticaria pigmentosa	<.0001	4.784	2.5	9.1
Urticaria	<.0001	23.111	8.4	63.3
Telangiectasia	0.0021	3.000	1.5	6.2
Rheumatic diseases	-			
Allergies	-			
Osteoporosis	<.0001	22.244	6.3	78.1
Liver affection	<.0001	34.536	11.7	102.2
Splenomegaly	<.0001	20.462*		
Fibromyalgia	0.0822	6.463	0.6	72.2
Eosinophilia	<.0001	19.890	4.3	91.7

For a number of symptoms odds ratios could not be calculated because data were not available.

* Odds ratio estimated.

**Table 4:** Odds ratios for the symptoms and findings collected in the questionnaire (independent variables) occurring in first-degree relatives, compared with the healthy control group.

	First-degree relatives vs. controls			
	p-value	Odds ratio	95%-confidence interval	
Female gender	0.6587	1.081	0.8	1.5
Smoker	0.0144	1.769	1.1	2.8
Abdominal pain	0.0037	2.073	1.3	3.4
Diarrhea	0.0002	2.425	1.5	3.9
Gastritis	0.0097	1.962	1.2	3.3
Meteorisms	0.0339	1.511	1.0	2.2
Nausea	0.0039	2.235	1.3	3.9
Non-cardiac chest pain	0.0719	1.981	0.9	4.2
Hypercholesterinemia	0.9907	1.002	0.7	1.5
Paresthesia/pain	0.0003	2.542	1.5	4.2
Tachycardia	0.0009	1.992	1.3	3.0
Flush	<.0001	3.807	1.9	7.5
Hot flash	<.0001	2.842	1.8	4.6
Blood pressure changes (acute)	0.0049	1.965	1.2	3.2
Thyroid dysfunction	0.5617	0.876	0.6	1.4
Diabetes mellitus	0.6074	1.194	0.6	2.4
Asthenia	<.0001	3.869	2.0	7.4
Fatigue	<.0001	2.619	1.7	4.1
Weight loss	0.1889	1.946	0.7	5.3
Angioedema	0.0531	2.286	1.0	5.4
Conjunctivitis	0.0008	2.324	1.4	3.8
Tinnitus	0.3104	1.267	0.8	2.0
Nasal congestion/rhinorrhea	0.0038	1.926	1.2	3.0
Pulmonary symptoms	0.0027	1.943	1.3	3.0
Headache	0.0120	1.719	1.1	2.6
Urticaria pigmentosa	0.2994	1.356	0.8	2.4
Urticaria	0.0111	3.481	1.3	9.6
Telangiectasia	0.7706	1.100	0.6	2.1
Rheumatic diseases	0.0410	3.578	1.0	13.2
Allergies	0.3105	1.200	0.8	1.7
Osteoporosis	0.0246	3.952	1.1	14.3
Liver affection	0.0552	2.953	0.9	9.4
Splenomegaly	0.1478	2.103*		
Fibromyalgia	0.0501	6.386	0.8	53.4
Eosinophilia	0.1653	0.518*		

For a number of symptoms odds ratios could not be calculated because data were not available.

* Odds ratio estimated.

### Spectrum of clinical and KIT affections within three selected pedigrees

**Figure 1:**
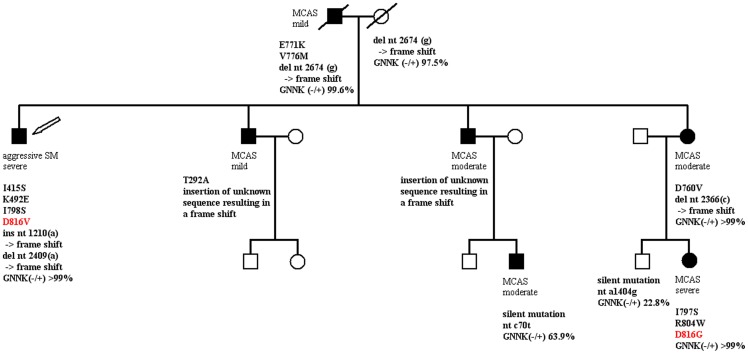
Pedigree of a family with a high familial loading of mast cell activation disease. Filled symbols indicate family members with either *systemic mastocytosis* (*SM*), according to WHO criteria, or *mast cell activation syndrome* (*MCAS*); open symbols denote clinically healthy family members. Squares, males; circles, females; symbols with a diagonal line through, deceased. *Mild*, *moderate* and *severe* indicate the clinical intensity of the mast cell activation disease. The arrow indicates the index patient. Bold type: genetic alterations detected in the tyrosine kinase, *KIT*. GNNK(–/+): ratio of the GNNK(–) over the GNNK(+) isoform (i.e., the amount of PCR amplification product without and with the tetrapeptide sequence glycine-asparagine-asparagine-lysine [GNNK]).

In the present study we investigated three selected families with a high familial loading of MCAD for variability in disease subtype and severity of disease, for different somatic mutations in the *KIT* gene at the level of mRNA and for differences in the expression of isoforms of the tyrosine kinase KIT. The MCAD subtype and the severity of the mediator-related symptoms varied, between the members of the families (Fig. 1 and Fig. S1). In addition variable genetic alterations in *KIT* were detected (Fig. 1 and Fig. S1). In two pedigrees, three members possessed a mutation at position 816 of the amino acid sequence of KIT (D816G and D816V, Fig. 1; D816V, Fig. S1A), whereas the other MCAD-affected family members had varying mutations at other sites of *KIT*. There was no obvious relation between the *KIT* mutations and the clinical severity of MCAD. However, in our patients with severe MCAD the expression intensity ratio for GNNK(–)/(+) (the KIT isoforms characterized by the presence or absence of the tetrapeptide sequence glycine-asparagine-asparagine-lysine (GNNK) in the extracellular part of the juxtamembrane region) was ≥ 95% (Fig. 1; Fig. S1).

## Discussion

The present study is the first systematic investigation of the familial occurrence of MCAD. Our data suggest that the prevalence of systemic MCAD is higher among relatives of MCAD patients than would be expected by chance, i.e., on the basis of the prevalence of MCAD in the general population. The prevalence of MCAD suspected by symptom self-report in first-degree relatives of patients with MCAD and in the German general population was approximately 46% and 17%, respectively (Table 2). Although familial occurrence due to shared environmental factors cannot be ruled out, it is likely that there is a significant genetic contribution to this familial occurrence. A preponderance of females was observed among those affected in all groups.

The 17% prevalence of MCAD suspected by symptom self-report in the German general population is probably due to the common occurrence of the MCAS subtype. Evidence has been presented that MCAS may be an underlying cause of various frequent clinical presentations; for example, in subsets of patients with fibromyalgia [18], [19] and irritable bowel syndrome [20]. Accordingly, the prevalence of MCAS, and therewith that of MCAD, was expected to be at least within the single-figure percentage range [3]. In previous studies we have presented indirect evidence for a potentially high prevalence of MCAD in the general population: (1) In human tissues, mast cells are the principal source of heparin. We found increased heparin levels in blood indicating a pathological increase in mast cell activity in approximately 50% of MCAD patients and and in 17% of the control subjects ([21] and references therein; unpublished data). (2) In our comparative analysis of *KIT* mutations in mast cells from patients with MCAD and control subjects we detected potentially functionally activating genetic alterations in 13 of 20 patients (65%) and in 3 of 20 control subjects (15%) [22]. In both studies those control subjects exhibiting an increased mast cell activity and *KIT* mutations, respectively, possibly represent individuals affected with MCAD at a subclinical stage. Thus, the prevalence of MCAD determined in this study fits well to these previous indirect estimations, suggesting that it is likely to reflect the true prevalence of the disease in the German population. A limitation of our study with regard to this conclusion may be that, in first-degree relatives and healthy control subjects, the diagnosis was made according to self-declared information. The sensitivity of the questionnaire to detect MCAD is high (reflected in the significantly increased prevalence of reported symptoms in MCAD compared with the control group) but it could not be reassessed whether the information about the absence or presence of relevant further diseases (differential diagnoses) were valid. Hence, the prevalence could be overestimated. In addition it cannot be excluded that recall bias in our controls has resulted in an overestimation of the prevalence of MCAD in the general population. Only 27% of the people whom we originally contacted completed the questionnaire and, since the purpose of the study and the disease were described in the addressing letter, affected people may have preferentially responded to our request. Therefore our estimation of the prevalence must be viewed with caution and should be taken as a stimulus to pursue more epidemiological studies in the future. For our study of familial aggregation, an overestimation of the true population prevalence had a conservative effect and therefore, does not affect the conclusions drawn from our observation of a significantly increased prevalence among first-degree relatives of patients.

In the three selected pedigrees, the MCAD subtype and the severity of the mediator-related symptoms varied between the members of the families. Similarly, in three of the five previous case reports on familial MCAD variability in the disease subtype, the severity of mediator-related symptoms, and the age at clinical onset within the MCAD sufferers has been described [9], [12], [23]. In four of those five case reports [10]–[13] functionally activating somatic point mutations were identified in mast cells which, however, were homotypic, i.e. identical between the affected family members. In contrast, in our pedigrees the genetic alterations detected in KIT varied between the affected family members as expected for genetic alterations occurring at the somatic level. The inherited genetic background leading to the familial occurrence of MCAD remains to be detected, and may, for example, involve operator and/or regulator genes of DNA synthesis, control and repair. The genetic mutations detected in the three families included mutations at position 816 of the amino acid sequence of KIT (D816G, Fig. 1; D816V, Fig. 1 and Fig. S1A). This finding is remarkable in that it disproves the longstanding (although unproven) assertion that the somatic nature of *KIT* ^D816V^ and related exon 17 mutations exclude heritability of the disease in the respective case [4], [24].

In the clinically affected but also in some of the clinically healthy family members of our three pedigrees multiple co-occurring mutations in the exons of the *KIT* gene were detected (the introns were not investigated). This finding supports our previous suggestion that the clinical presentation of MCAD, i.e. subtype, severity and therapy responsiveness, is not determined by one *KIT* mutation alone but by multiple co-occuring mutations in *KIT* and/or other relevant genes [3], [22]. In fact, there is accumulating evidence for the presence of disease-relevant multiple co-occurring mutations in *KIT* [12], [17], [22], [25], [26] as well as of a combination of *KIT* mutations with mutations in other genes (e.g., JAK2, TET2, DNMT3A, ASXL1, CBL, U2AF1, SRSF2, MS4A2; [11], [27]–[31]). In accordance with this idea that a mutation on its own does not define the clinical phenotype of MCAD, there was no obvious relation between the *KIT* mutations detected in the three families and the severity of MCAD of the affected family members in the present study. However, it has been reported that the predominance of a certain KIT isoform, nameley of the GNNK(-) isoform which is characterized by the absence of the tetrapeptide sequence glycine-asparagine-asparagine-lysine (GNNK) in the extracellular part of the juxtamembrane region and which shows tumorigenic potency, may contribute to, or reflect a pathological increase in, the activation of affected mast cells [3]. In the present three pedigrees the expression intensity ratio for GNNK(–)/(+) isoforms was ≥ 95% in all patients with severe MCAD (Fig. 1; Fig. S1) which is in agreement with previous data [3]. Although the reason for the predominant expression of the GNNK(–)-isoform in those patients is still unknown, its presence may support the diagnosis MCAD [3].

In conclusion, our data provide evidence for common familial occurrence of MCAD. This makes the future application of systematic molecular genetic studies, such as genome-wide association studies, to MCAD very promising. Together with findings of previous studies, which suggested that almost all *KIT* mutations were somatic rather than germline (reviewed in [3]), our present data support the idea that in familial cases (i.e., the majority of MCAD) mutated disease-related operator and/or regulator genes could be responsible for the development of somatic mutations in KIT and other proteins involved in the regulation of mast cell activity [3]. Accordingly, the immunohistochemically different subtypes of MCAD (MCAS and SM) should be more accurately regarded as varying presentations of a common generic root process of mast cell dysfunction, than as distinct diseases [1], [3].

## Supporting Information

Figure S1 **Pedigrees of two families (A, B) with high familial loading of mast cell activation disease.** Filled symbols indicate family members with either *systemic mastocytosis* (*SM*) according to WHO criteria, or *mast cell activation syndrome* (*MCAS*); open symbols denote clinically healthy family members. Squares, males; circles, females. *Mild*, *moderate*, and *severe* indicate the clinical intensity of the mast cell activation disease. The arrow indicates the index patient. Bold type: genetic alterations detected in the tyrosine Kinase, *KIT*. GNNK(−/+): ratio of the GNNK(−) over the GNNK(+) isoform (i.e., the amount of PCR amplification product without and with the tetrapeptide sequence glycine-asparagine-asparagine-lysine [GNNK]).
(SDR)

Table S1 **Characteristics of the study population by participant status.**
(DOC)

Table S2 **Questionnaire to diagnose** ***mast cell activation disease*** **from clinical findings.** MCAD was diagnosed after exclusion of relevant differential diagnoses, if (1) 11 or more items were applicable or (2) if potentially mast cell mediator-related symptoms occurred in five or more different organs and/or tissues.
(DOC)

Table S3 **Sequences of the forward and reverse primers used for PCR amplification.**
(DOC)
