# Supplementary material for: Familial Occurrence of Systemic Mast Cell Activation Disease
Source: PLoS One. 2013 Sep 30;8(9):e76241. doi: 10.1371/journal.pone.0076241 (PMC3787002; doi:10.1371/journal.pone.0076241)
Supplement: Table S1 — Characteristics of the study population by participant status. (DOC) [file pone.0076241.s002.doc]

**Supporting Table S1.** Characteristics of the study population by participant status.
