# Supplementary material for: Familial Occurrence of Systemic Mast Cell Activation Disease
Source: PLoS One. 2013 Sep 30;8(9):e76241. doi: 10.1371/journal.pone.0076241 (PMC3787002; doi:10.1371/journal.pone.0076241)
Supplement: Table S2 — Questionnaire to diagnose mast cell activation disease from clinical findings. MCAD was diagnosed after exclusion of relevant differential diagnoses, if (1) 11 or more items were applicable or (2) if potentially mast cell mediator-related symptoms occurred in five or more different organs and/or tissues. (DOC) [file pone.0076241.s003.doc]

**Supporting Table S2.** Questionnaire to diagnose *mast cell activation disease* from clinical findings.

MCAD was diagnosed after exclusion of relevant differential diagnoses, if (1) 11 or more items were applicable or (2) if potentially mast cell mediator-related symptoms occurred in five or more different organs and/or tissues.

**Origin (place of birth/region/country) of the grandparents**

paternal grandfather ________________ grandmother ________________

place of birth/region/country place of birth/region/country

maternal grandfather ________________ grandmother ________________

place of birth/region/country place of birth/region/country

Age (year of birth)

Body height (cm)

Body weight (kg)

***Please mark applicable***

Gender female 🞎 male 🞎

Smoker 🞎 Non smoker 🞎

***I have, or have had, the following symptoms during the least 2 years***

Recurring or continuing burning and/or crampy abdominal pain of unknown cause 🞎

Recurring or continuing diarrhea of unknown cause 🞎

Recurring gastritis or gastric ulcers of unknown cause 🞎

Frequently intense meteorism (independent of the composition of food) 🞎

Recurring nausea of unknown cause 🞎

Episodically paroxysmally occuring burning and/or choking pain in the chest 🞎

Increased level of cholesterol in the blood 🞎

Occasional or continuing paresthesia (burning, pins and needles, numbness) and/or pain, which does not respond to treatment with analgesics 🞎

Tachycardia or allodromy 🞎

Paroxysmal intense redness and warmth in a confined area of the body (flush) 🞎

Hot flashes 🞎

Episodically paroxysmally occuring sudden changes in blood pressure (fall or elevation) 🞎

Thyroid dysfunction

hyperthyroidism 🞎

hypothyroidism 🞎

Diabetes mellitus 🞎

Asthenia 🞎

Fatigue 🞎

Episodically weight loss 🞎

Episodically swelling of the face 🞎

Chronic conjunctivitis (reddened and/or itchy and/or dry eyes) 🞎

Tinnitus 🞎

Rhinorrhea or chronic nasal congestion 🞎

Non-allergic respiratory ailments such as asthma, compulsion to clear one´s throat, titillative feeling in the respiratory tract and/or shortness of breath during routine tasks 🞎

Sudden, migraine-like headache 🞎

Skin alteration in the form of reddish brown, possibly elevated spots 🞎

urticaria 🞎

an increase in the number of fine dot-shaped dilated blood vessels (telangiectasia) 🞎

Rheumatic disease (please indicate the exact diagnosis, if known) 🞎

Allergies (please indicate trigger, if known) 🞎

Suspicious results of a laboratory test and/or findings (e.g., findings in gastroscopy, coloscopy, ultrasonic scan, ECG, bone densitometry)

I have the following disease:

*Porphyria* 🞎

*Hereditary jaundice (Hyperbilirubinemia)* 🞎

*Fabry disease* 🞎

*Helicobacter-positive gastritis* 🞎

*Infectious intestinal inflammation* 🞎

*Parasitical intestinal infection* 🞎

*Ulcerative Colitis* 🞎

*Crohn’s disease* 🞎

*Primary celiac disease (gluten sensitive enteropathy)* 🞎

*Lactose intolerance* 🞎

*Fructose intolerance* 🞎

*Microscopic colitis* 🞎

*Amyloidosis* 🞎

*Liver inflammation (hepatitis)* 🞎

*Cholelithiasis* 🞎

*Neuroendocrine tumor (carcinoid, pheochromocytoma, VIPoma,*

*gastrinoma, insulinoma, glucagonoma, somatostatinoma)* 🞎

*Food allergy* 🞎

*Hypereosinophilic syndrome* 🞎

*Hereditary angioedema* 🞎

*Autoimmune vasculitis* 🞎

*Other (please indicate)* 🞎

Current intake of medication:
