# Supplementary material for: Familial Occurrence of Systemic Mast Cell Activation Disease
Source: PLoS One. 2013 Sep 30;8(9):e76241. doi: 10.1371/journal.pone.0076241 (PMC3787002; doi:10.1371/journal.pone.0076241)
Supplement: Table S3 — Sequences of the forward and reverse primers used for PCR amplification. (DOC) [file pone.0076241.s004.doc]

#### Supporting Table S3. Sequences of the forward and reverse primers used for PCR amplification

| **Primer sequence** | **Location in the coding sequence of *KIT*** | **Annealing temperature** |
| --- | --- | --- |
| forward primer: 5´-CGAGAGCTGGAACGTGGACC -3´  reverse primer: 5´-*CATTTAGGTGACACTATAGA*CTCCTGTAGTTTAGTCTGCTG -3´  reverse primer: 5´-*CATTTAGGTGACACTATAGA*CTCCTGTAGTTTAGTCTGAC -3´ | 5´-UTR -49– -30  754–771  752–771 | 57°C |
| forward primer: 5´-*TAATACGACTCACTATAGGG*GAAAAGAGAAAACAGTCAGCAG -3´  forward primer: 5´-*TAATACGACTCACTATAGGG*GAAAAGAGAAAACAGTCAGAC -3´  reverse primer: 5´-CTCGTTAGATGAAGTTCACTTAC -3´ | 738–756  738–758  1120–1142 | 54°C |
| forward primer: 5´-GCAGTGGATCTATATGAACAG -3´  reverse primer: 5´-*CATTTAGGTGACACTATAGA*GGATTTGCTCTTTGTTGTTAC -3´  reverse primer: 5´-*CATTTAGGTGACACTATAGA*GATGGATTTGCTCTTTAAATGC -3´ | 1038–1058  1529–1550  1519–1552 | 54°C |
| forward primer: 5´-*TAATACGACTCACTATAGGG*GTAACAACAAAGAGCAAATCC -3´  forward primer: 5´-*TAATACGACTCACTATAGGG*CATTTAAAGAGCAAATCCATC -3´  reverse primer: 5´-*CATTTAGGTGACACTATAGA*CTCATTAGTACTATCGCTGCAG -3´  reverse primer: 5´-*CATTTAGGTGACACTATAGA*GTACTCATTAGTACTATCGCAG -3´ | 1529–1549  1520–1552  2139–2160  2139–2163 | 54°C |
| forward primer: 5´-*TAATACGACTCACTATAGGG*CAAAGGAGTCTTCCTGCAGC -3  forward primer: 5´-*TAATACGACTCACTATAGGG*CAAAGGAGTCTTCCTGCGATAG -3  reverse primer: 5´-CTGCTTCCTAAAGAGAACAG -3´ | 2126–2145  2126–2150  2584–2603 | 54°C |
| forward primer: 5´-GACTACCTGTGAAGTGGATG -3´  reverse primer: 5´-CAGAAAGACAGGATTGCAGTG -3´ | 2489–2508  3´-UTR +112– +132 | 57°C |

#### Italic letters: sequence of the universal primers T7 and SP6, respectively.

Please note that those constructs which are designed to recognize minus splice variants lack the corresponding nucleotide sequence of the reference sequence.
